# Is there any putative mediatory role of inflammatory markers on the association between ultra-processed foods and resting metabolic rate?

**DOI:** 10.3389/fnut.2022.932225

**Published:** 2022-10-13

**Authors:** Niki Bahrampour, Farideh Shiraseb, Sahar Noori, Cain C. T. Clark, Khadijeh Mirzaei

**Affiliations:** ^1^Department of Nutrition, Science and Research Branch, Islamic Azad University (SRBIAU), Tehran, Iran; ^2^Department of Community Nutrition, School of Nutritional Sciences and Dietetics, Tehran University of Medical Sciences (TUMS), Tehran, Iran; ^3^Centre for Intelligent Healthcare, Coventry University, Coventry, United Kingdom

**Keywords:** resting metabolic rate, ultra-processed foods (UPFs), inflammation, high-sensitivity C-reactive protein (hs-CRP), monocyte chemoattractant protein (MCP-1)

## Abstract

The resting metabolic rate (RMR) represents the largest component of total daily energy expenditure. The sale of ultra-processed foods (UPF) is increasing globally; however, UPF can have many adverse effects, including increasing inflammatory markers and altering RMRs. This cross-sectional study included 285 healthy overweight and obese women. Anthropometric measurements were evaluated using a bioelectrical impedance analyzer InBody 770 scanner. High-sensitivity C-reactive protein (hs-CRP), plasminogen activator-1 (PAI-1), monocyte chemoattractant protein (MCP-1), and interleukin-1 beta (IL-1β) blood levels were measured after a 12-h fasting. Indirect calorimetry was used to evaluate the RMR by using the Weir equation, and RMR deviation (RMR estimated - RMR actual), RMR per body mass index (BMI), and free fat mass (FFM) were estimated. A validated food frequency questionnaire (FFQ) was used, and seven groups of UPFs were extracted based on the NOVA method. A negative association between the RMR [β = −0.159, 95% confidence interval (CI): −0.471, −0.052, *P* = 0.044], RMR per BMI (β = −0.014, 95% CI: −0.025, −0.006, *P* = 0.036), and RMR per FFM (β = −0.241, 95% CI: −0.006, −0.000, *P* = 0.041) using the NOVA score was observed after adjusting for confounders. This association disappeared after inclusion of each inflammatory marker. All the markers may inversely mediate the relationship between the mentioned variables and the NOVA score. hs-CRP and MCP-1 also had a negative effect on the relationship between the NOVA score and RMR deviation. Finally, UPF intake is likely related with the RMR, mediated through changes in the production of hs-CRP, PAI-1, MCP-1, and IL-1β.

## Introduction

Knowledge of the resting metabolic rate (RMR) is necessary because it represents the largest component of total daily energy expenditure and has ramifications for nutrition support. Obesity and alterations in the RMR have a close link (1); indeed, obesity is one of the most important reasons for decreases in metabolism, and given that the prevalence of obesity and overweight has doubled in more than 70 countries since 1980 (50% of women are overweight and obese), an important consideration is the concomitant change in the RMR (1–3). The RMR is defined as the energy requirement of humans when they are awake in a normal temperature room, after food absorption, and not engaging in any activity for 12 h (4). Many previous studies have reported that the RMR is usually impacted by sex disparities, obesity, age, and racial/ethnic differences (4). For example, the RMR has been shown to be higher in men than in women due to their muscle mass (5), while aging has also been shown to affect the RMR (6). Dietary intake represents another factor that can affect the RMR; for instance, a recent study suggested that consuming protein in obese persons could contribute to an increase in resting energy expenditure (REE) (7). Lichtenbelt et al. found that consumption of polyunsaturated fatty acids to a greater extent than saturated fatty acids can result in an increasing RMR due to a higher oxidation rate (8). Other studies have reported that low-carbohydrate (<45% energy from carbohydrate) and low glycemic foods facilitate a RMR reduction in weight loss plans, largely due to more availability and the presence of endocrine mediators in anabolic and catabolic pathways (9). In order to identify processed foods, the NOVA classification system represents a novel tool for gaining insights into the epidemic (10). Foods are separated into unprocessed foods, processed culinary ingredients, processed foods, and ultra-processed foods (UPFs). UPFs refer to foods that are produced through industrial processes like hydrogenation, hydrolysis, extruding, molding, reshaping, and frying, and adding flavors, colors, emulsifiers, humectants, non-sugar sweeteners, and other additives (11); sodas, breakfast cereals, packaged cakes, and pre-prepared frozen foods are examples of this group.

There are serious concerns about ultra-processed food (UPF) intake and obesity (11). The sale of UPFs is increasing all over the world (to 43.7% until 2013) and has replaced fresh foods in low- and middle-income countries (12, 13). The 2015–2020 dietary guidelines for Americans suggest people increase high-quality dietary patterns for preventing chronic diseases (10). UPFs are made with various additives (salt, sugar, fat, etc.) and are low in dietary fiber and micronutrients (14), and the extant literature suggests a positive relationship between obesity, inflammation, and consumption of UPFs (14). In addition, recent studies have shown that a higher intake of UPFs is associated with lowering RMRs, slowing satiation, and weight gain, through lower protein, antioxidants, fiber, and higher energy density, and refined grain content present in them (15). Furthermore, unhealthy UPFs can interrupt gut–brain signaling, change vascular stiffness, and increase inflammatory markers (16, 17). Plasminogen activator-1 (PAI-1) is secreted by adiposity tissues in the blood and may have a negative effect on RMRs (18, 19). Moreover, UPF intake can increase the serum level of monocyte chemoattractant protein (MCP)-1 (20), while another inflammatory marker, interleukin-6 (IL-6), is decreased in a diets abundant in ω-3 polyunsaturated fatty acids (PUFAs) (21). Anti-inflammatory diets, such as the Mediterranean diet, which has a high amount of vegetables and fruit, fiber, and vitamins C and E, are related to decreased serum levels of pro-inflammatory biomarkers, like high-sensitivity C-reactive protein (hs-CRP) (22). Indeed, nutrients in these diets are low in UPF and may be important in regulating the immune system and changes in RMR (18).

To the best of our knowledge, no study has evaluated the impact of inflammatory markers on the relationship between RMR and UPF intake, using the NOVA classification, in Iran. Therefore, this study was designed to assess the possible mediatory role of inflammatory markers in the association between ultra-processed foods and the resting metabolic rate.

## Materials and methods

### Study population

This cross-sectional study was performed with 285 overweight and obese women, who were referred to healthcare centers in Tehran, Iran. Participants were selected using multistage random sampling. The inclusion criteria were given as follows: aged between 18 and 48 years, body mass index (BMI) scores 25–29.9 kg/m^2^ for overweight and 30 kg/m^2^ for obesity, and without any chronic diseases, like hypertension, kidney or liver disorders, and cardiovascular diseases, which was ascertained by a face-to-face anamnesis interview. Women who were smokers; were alcohol consumers; had prescribed medication; and were pregnant, breastfeeding, or in menopausal stage; or had a history of weight loss in the last 6 months were excluded. In addition, only participants with a total energy intake between 800 and 4,200 kcal/d were included (23).

### Experimental methods

This study was conducted according to the guidelines of the Declaration of Helsinki, and all procedures involving human subjects/patients were approved by the ethics committee of Tehran University of Medical Sciences (TUMS) with the following identification: IR.TUMS.VCR.REC.1398.463. Written informed consent was obtained from all patients prior to participation.

### Anthropometric measurements

Weight, BMI, and body fat mass (BFM) were measured using a multi-frequency bioelectrical impedance analyzer (BIA) InBody 770 scanner (Inbody Co., Seoul, Korea), where the women wore light clothes, without any metal subjects, and in accordance with manufacturer’s protocols. Anthropometric measurements were conducted between 8 and 9 a.m., after 12 h of fasting. The participants were asked to refrain from any non-habitual physical activity for the preceding 72 h. The test–re-test reliability of BIA in our laboratory was *r* = 0.98. BFM (kg), free fat mass (FFM) (kg), and fat mass index (FMI) (kg/m^2^) were extracted. The height of the participants was measured with a Seca 216 stadiometer, to the nearest 0.1 cm, with the participants in a standing position and unshod. The arm muscle circumference (cm) of the participants were also measured according to standard kinanthropometrist guidelines.

### Serum biochemical measuring

After fasting for 12 h, blood samples were drawn from the participants. The samples of blood were collected in parent tubes containing 0.1 EDTA, and tests were analyzed utilizing the Auto-Analyzer BT 1500 (Selectra 2; Vital Scientific, Spankeren, Netherlands). hs-CRP, PAI-1, MCP-1, and interleukin-1 beta (IL-1β) levels were measured by an immunoturbidimetric test using the Pars Azmoon kit (Pars Azmoon Inc., Tehran, Iran). Serum levels of high-density lipoprotein cholesterol (HDL-C), fasting blood sugar (FBS), low-density lipoprotein cholesterol (LDL-C), total cholesterol (TC), insulin, and triglyceride (TG) were also measured. Insulin resistance was assessed as HOMA-IR (homeostasis model assessment-estimated insulin resistance) = [glucose] (mmol/l) × [insulin] (μU/ml)/22.5 (24).

### Energy expenditure measurements

Indirect calorimetry (IC) is considered an accurate method of determining the RMR and was used to evaluate the RMR after an overnight fast. Respiratory gases were gathered using the MetaLyzer 3B-R3 (Cortex Biophysik GmbH, Leipzig, Germany) spirometer. The participants were asked to refrain from the consumption of caffeine and vigorous activity for a day prior to RMR measurements. The participants were asked to sleep in a silent place in a temperature between 24 and 26°C the night before the test and be free from emotional stress. Gaseous exchange was analyzed in the last 20 min of the resting time and during a minimum of 5 consecutive minutes in a steady-state condition. The RMR was measured using the Weir equation: RMR = [O_2_ consumed (liter) × 3.941 + produced Co_2_ (liter) × 1.11] × 1,440 min/d by assessing the amount of CO_2_ produced and O_2_ consumed through a one-way valve between the participants and the machine (25). RMR deviation was computed as RMR estimated - RMR actual (26). The RMR was also calculated per body surface area (BSA) using the Du Bois formula, BMI, and FFM (which were derived from BIA results) (27–29).

### Dietary intake and NOVA score assessment

A validated food frequency questionnaire (FFQ), with 147 items, was used to assess the average food consumption of the participants over the past year (30). All dietary intakes, including foods and beverages, were converted to grams (31) and entered into Nutritionist IV software (version 7.0; N-Squared Computing, Salem, OR). In order to classify processed foods, the NOVA method was used (32). A total of 37 UPF items were categorized into seven food groups, and then the mean daily intake of each of seven UPF groups was divided by the total daily intake of UPF and multiplied by 100. These groups included non-dairy beverages (Cola, nectar drink, and instant coffee), cookies–cakes [cookies, biscuit, pastries (creamy and non-creamy), cake, pancake, doughnut, industrial bread, toasted bread, noodles, and pasta), dairy beverages [ice cream (non-pasteurized), ice cream (pasteurized), chocolate milk, and cocoa milk], potato chips—salty snacks [chips (crisps), crackers, and cheese puff], processed meat fast foods [burger, sausage, bologna, and pizza], oil sauces (margarine, ketchup, and mayonnaise), and sweets (jam, rock candy, candies, chocolates, sweets, nogal, sohan, Gaz, and sesame halva) (32).

### Other variables

The physical activity (PA) of the participants was also evaluated *via* the short form of the International Physical Activity Questionnaire (IPAQ) (33). The metabolic equivalent (MET) and MET-minutes per week (MET-min/wk) were ascertained by summing the activity hours per week.

### Statistical analysis

The normality of data was evaluated using a Kolmogorov–Smirnov test. General characteristics of the participants within different tertiles of the NOVA score were described as mean ± standard deviation (SD) for quantitative variables and frequency (%) for categorical variables. Chi-square tests and one-way analysis of variance (ANOVA) and analysis of covariance (ANCOVA) were used for categorical variables and dietary intake in order to compare the differences among tertiles of NOVA scores. The NOVA score was divided into three groups (T1 < 383.681, 383.681 < T2 < 467.713, and T3 > 467.713). Bonferroni *post hoc* tests were used to investigate differences between tertiles. Linear regression was also used to investigate the relationship between outcomes and exposure in crude and two adjusted models and the role of inflammatory markers. Model 1 was adjusted for age, energy intake, BMI, physical activity, job status, and supplement intake. In model 2, additional controlling for legumes and vegetables was conducted. The results are shown as a beta (β) with 95% confidence intervals (95% CI). Pearson correlation was utilized to discern the association between inflammatory markers, RMR, and NOVA score. All analyses were conducted using Statistical Package for Social Sciences) SPSS) version 25 software (SPSS Inc., Chicago, Illinois), where *P* < 0.05 was considered statistically significant.

## Results

### Study population characteristics

As shown in Supplementary Table 1, according to NOVA screening, most of the UPF intakes were *via* non-dairy beverages (39.12), and the smallest contribution was from oils and sauces (4.33%). General characteristics of the participants across NOVA score tertiles are shown in Table 1. The mean (SD) of weight, FFM, and BMI was 80.92 (12.39) kg, 46.76 (5.59) kg, and 31.02 (4.29) kg/m^2^, respectively. In addition, the total mean (SD) of serum levels of hs-CRP, IL-1β, MCP-1, and PAI-1 was 4.31 (4.65), 2.73 (0.94), 50.69 (92.46), and 16.10 (29.92) mg/dl, respectively. A total of 142 women had a moderate income, and 148 were taking dietary supplements. Age and height were significantly different among NOVA tertiles (*P* < 0.05) in the crude model. After adjusting for confounding variables, such as age, BMI, PA, and energy intake, hs-CRP, and age were all significant (*P* < 0.05) (Table 1).

**TABLE 1:** Distribution of general variables of the participants among tertiles of UPF intake.

Quantitative variables	T1 (<383.681)	T2 (383.681–467.713)	T3 (>467.713)	*P*-value	P*-value
Age (year)	36.48 ± 9.13	38.75 ± 8.77 ^b^	34.86 ± 9.35 ^c^	**0.003**	**0.004**
Weight (kg)	81.95 ± 12.38	79.88 ± 10.97	81.66 ± 13.32	0.33	0.22^a^
Height (cm)	161.57 ± 5.88	160.11 ± 5.88	161.76 ± 5.79	**0.04**	0.91^a^
WC (cm)	97.28 ± 17.05	97.13 ± 12.69	97.95 ± 16.05	0.93	0.24^a^
BFM (kg)	34.93 ± 8.39	33.83 ± 7.80	35.42 ± 9.88	0.32	0.25^a^
FFM (kg)	47.01 ± 5.93	46.21 ± 5.44	46.26 ± 5.61	0.32	0.41^a^
BMI (kg/m^2^)	31.36 ± 4.11	31.19 ± 3.98	31.25 ± 4.79	0.94	0.31^a^
Arm muscle circumference (cm)	28.64 ± 4.56	28.22 ± 1.98	28.11 ± 2.83	0.40	0.21^a^
FMI (kg/m^2^)	13.42 ± 3.16	13.31 ± 3.23	13.61 ± 3.79	0.78	0.45^a^
PA (METs-hour-week)	1454.73 ± 2424.86	825.17 ± 901.18	1365.72 ± 2653.36	0.09	0.15
Hs-CRP (mg/dl)	4.30 ± 4.62	4.21 ± 4.64 ^b^	4.48 ± 4.77 ^c^	0.94	<**0.001**
IL-1 (mg/dl)	2.58 ± 0.89	2.74 ± 1.02	2.84 ± 0.92	0.64	0.95
MCP-1 (mg/dl)	57.51 ± 94.78	54.33 ± 109.98	36.96 ± 54.65	0.38	0.27
PAI-1 (mg/dl)	20.26 ± 39.58	14.20 ± 24.73	13.31 ± 20.37	0.40	0.33
Insulin	1.20 ± 0.24	1.24 ± 0.23	1.19 ± 0.19	0.41	0.41
HOMA_IR index	3.24 ± 1.34	3.58 ± 1.38	3.14 ± 1.00	0.07	0.05
**Qualitative variables**					
Supplementation intake n (%)				0.31	0.05
Yes	58 (36.7)	47 (29.7)	53 (33.5)		
No	51 (29)	61 (34.7)	64 (36.4)		
Income status n (%)				0.58	0.18
Weak	33 (37.5)	31 (35.2)	24 (27.3)		
Moderate	60 (33.0)	61 (33.5)	61 (33.5)		
High	35 (32.7)	31 (29.0)	41 (38.3)		
Marital status n (%)				0.27	0.88
Single	35 (32.1)	31 (28.4)	43 (39.4)		
Married	92 (33.6)	96 (35.0)	86 (31.4)		

SD, Standard deviation; METs, Metabolic Equivalents; PA, physical activity; BMI, body mass index; WC, waist circumference; BFM, body fat mass; FFM, fat free mass; FMI, fat mass index; hs-CRP, high sensitive- C reactive protein; IL-1, interleukin-1; MCP-1, monocyte chemoattractant protein-1; PAI-1, plasminogen activator inhibitor- 1, HOMA-IR index, insulin resistant.

Quantitative variables were showed by means ± SD and qualitative variables were showed by number (percentage).

*P*-values resulted from one-way ANOVA analysis and chi-square test. *P*-value < 0.05 was considered significant with bold font.

*P-values resulted from ANCOVA analysis and were adjusted for age, BMI, PA, and energy intake.

^*a*^Variables just adjusted for age, energy intake, and PA and BMI was considered as collinear.

Bonferroni *Post hoc* test was used to investigate differences between tertiles. ^b,c^Between two means is significant difference at 0.05.

### Dietary intake across NOVA score tertiles

The dietary intake of subjects between NOVA tertiles is shown in Table 2. All of the food groups and components were statistically different among NOVA tertiles, except whole grain, vitamin D, and trans fat (*P* < 0.05). After adjusting for energy, vegetables (*P* = 0.003), legumes (*P* = 0.04), linolenic acid (*P* = 0.02), total fiber (*P* < 0.001), beta-carotene (*P* = 0.01), vitamin B1 (*P* = 0.002), B6 (*P* < 0.001), B9 (*P* < 0.001), biotin (*P* = 0.01), magnesium (*P* = 0.002), copper (*P* = 0.008), and chromium (*P* = 0.009) remained significantly different.

**TABLE 2:** Intake of UPF, food groups, and nutrients according to the UPF tertiles in overweight and obese women.

Variables	Total mean ± SD	T1 (<383.681)	T2 (383.681–467.713)	T3 (>467.713)	*P*-value	P*-value
Energy (kcal)	2633.28 ± 809.43	2916.67 ± 654.47	2267.60 ± 712.43	2713.37 ± 904.86	<**0.001**	–
**NOVA food groups**						
Non-dairy beverages (g/d)	177.35 ± 93.22	124.06 ± 25.54	157.15 ± 27.64	251.24 ± 126.71	<**0.001**	<**0.001**
Cookies-cakes (g/d)	98.91 ± 44.20	75.57 ± 25.62	97.28 ± 28.00	124.06 ± 57.16	<**0.001**	<**0.001**
Dairy beverages (g/d)	47.83 ± 27.95	37.47 ± 18.48	46.62 ± 22.11	59.47 ± 35.79	<**0.001**	<**0.001**
Potato chips- salty snack (g/d)	22.10 ± 13.89	17.35 ± 9.09	22.65 ± 10.16	26.34 ± 18.85	<**0.001**	<**0.001**
Processed meat- fast food (g/d)	41.13 ± 25.42	28.40 ± 12.60	40.23 ± 14.20	54.88 ± 35.16	<**0.001**	<**0.001**
Oil-sauce (g/d)	18.26 ± 8.72	16.76 ± 8.54	17.86 ± 7.41	20.19 ± 9.76	**0.005**	**0.005**
Sweet (g/d)	36.86 ± 24.06	30.67 ± 15.11	36.91 ± 17.18	43.03 ± 33.87	<**0.001**	<**0.001**
**Food groups**						
Fruits (g/d)	528.90 ± 338.16	605.77 ± 317.15	466.28 ± 317.37	513.25 ± 370.04	**0.01**	0.34
Vegetables (g/d)	433.57 ± 263.25	526.61 ± 264.20	382.92 ± 226.81	385.49 ± 275.07	<**0.001**	**0.003**
Nuts (g/d)	14.37 ± 16.18	17.82 ± 17.78	11.44 ± 14.35	13.79 ± 15.69	**0.01**	0.51
Meat (g/d)	64.57 ± 50.17	67.37 ± 40.97	54.08 ± 41.67	73.51 ± 65.01	**0.02**	0.25
Whole grains (g/d)	7.58 ± 10.41	9.14 ± 11.23	6.76 ± 9.01	6.74 ± 10.83	0.17	0.36
Legumes (g/d)	52.69 ± 41.27	63.43 ± 49.57	45.83 ± 35.76	48.31 ± 34.08	**0.005**	**0.04**
**Nutrients**						
Protein (g/d)	91.31 ± 31.45	101.24 ± 25.30	78.89 ± 28.96	93.71 ± 35.30	<**0.001**	0.11
Total fat (g/d)	95.13 ± 35.17	103.81 ± 34.89	82.52 ± 29.36	99.01 ± 37.40	<**0.001**	0.43
Carbohydrate (g/d)	372.45 ± 124.59	417.51 ± 104.94	318.88 ± 113.02	380.60 ± 134.31	<**0.001**	0.10
SFA (g/d)	28.40 ± 11.54	30.86 ± 11.41	24.76 ± 10.29	29.58 ± 12.03	<**0.001**	0.62
Cholesterol (mg/d)	264.06 ± 113.12	276.57 ± 105.30	242.75 ± 104.47	272.77 ± 126.13	**0.03**	0.37
PUFA (g/d)	20.08 ± 9.56	22.58 ± 10.51	17.40 ± 8.31	20.23 ± 9.08	<**0.001**	0.71
Linolenic acid (g/d)	1.21 ± 0.66	1.43 ± 0.74	1.04 ± 0.58	1.16 ± 0.60	<**0.001**	**0.02**
Trans fat (g/d)	0.0007 ± 0.002	0.001 ± 0.003	0.0006 ± 0.001	0.0005 ± 0.001	0.09	0.12
Total fiber (g/d)	47.34 ± 21.36	57.26 ± 21.37	40.35 ± 19.20	44.33 ± 19.79	<**0.001**	<**0.001**
Beta-carotene (mg/d)	5123.54 ± 3403.75	5983.44 ± 3233.19	4811.75 ± 3878.53	4568.82 ± 2879.15	**0.001**	**0.01**
C (mg/d)	188.40 ± 116.76	215.84 ± 103.63	164.99 ± 104.12	184.18 ± 134.97	**0.002**	0.33
D (μg)	1.95 ± 1.55	2.09 ± 1.84	1.82 ± 1.51	1.96 ± 1.25	0.377	
E (mg/L)	17.01 ± 9.05	18.75 ± 9.46	15.49 ± 8.54	16.78 ± 8.89	**0.01**	0.60
B1 (mg/d)	2.13 ± 0.73	2.44 ± 0.67	1.84 ± 0.66	2.12 ± 0.74	<**0.001**	**0.002**
B2 (mg/d)	2.27 ± 0.87	2.47 ± 0.77	2.01 ± 0.82	2.33 ± 0.95	<**0.001**	0.72
B6 (mg/d)	2.19 ± 0.75	2.51 ± 0.63	1.93 ± 0.70	2.14 ± 0.81	<**0.001**	<**0.001**
Folate (μg/d)	620.27 ± 192.93	706.78 ± 171.92	542.35 ± 173.64	611.01 ± 196.96	<**0.001**	<**0.001**
Biotin (mg/d)	38.25 ± 16.81	43.60 ± 14.44	34.89 ± 14.78	36.24 ± 19.54	<**0.001**	**0.01**
Sodium (mg/d)	4482.75 ± 1757.72	4853.93 ± 1702.71	4032.31 ± 1367.83	4559.15 ± 2048.83	**0.001**	0.77
Potassium (mg/d)	4509.82 ± 1727.13	5089.18 ± 1580.68	3934.96 ± 1545.09	4500.85 ± 1854.49	<**0.001**	0.16
Magnesium (mg/d)	475.67 ± 171.57	546.11 ± 159.40	410.33 ± 149.70	470.04 ± 177.79	<**0.001**	**0.002**
Copper (mg/d)	2.02 ± 0.75	2.32 ± 0.64	1.72 ± 0.65	2.00 ± 0.82	<**0.001**	**0.008**
Chromium (mg/d)	0.12 ± 0.10	0.15 ± 0.12	0.10 ± 0.07	0.10 ± 0.08	<**0.001**	**0.009**

SD, Standard deviation; SFA, saturated fatty acids; PUFA, polyunsaturated fatty acids.

*P*-values are resulted from ANOVA analysis. *P*-value < 0.05 was significant with bold font.

*P-values presented resulted from ANCOVA analysis and were adjusted for energy except energy intake.

### Distribution of resting metabolic rate measurements among NOVA score tertiles

According to Table 3, the mean RMR was not significantly different between the tertiles of NOVA in the crude model. Deviation from the estimated normal RMR was significantly different in model 2 (*P* = 0.04).

**TABLE 3:** The distribution of RMR subcategories between UPF intake tertiles.

Variables	T1 (<383.681)	T2 (383.681–467.713)	T3 (>467.713)	*P*-value	P*-value
**RMR (kcal)**					
Crude	1576.69 ± 280.39	1564.44 ± 238.68	1592.00 ± 259.98	0.772	
Model 1^b^	1602.81 ± 30.98	1542.07 ± 31.25	1530.21 ± 32.54		0.315
Model 2^c^	1560.92 ± 31.46	1598.37 ± 30.93	1525.47 ± 32.72		0.290
**RMR deviation (kcal)**					
Crude	-9.53 ± 12.58	-7.23 ± 13.49	-7.97 ± 11.82	0.429	
Model 1	-5.49 ± 1.62	-10.88 ± 1.64	-9.75 ± 1.71		0.066
Model 2	-9.77 ± 1.64	-5.75 ± 1.61	-10.68 ± 1.71		**0.045**
**RMR per BSA (kcal)**					
Crude	844.85 ± 115.94	855.77 ± 120.64	857.10 ± 105.98	0.720	
Model 1	827.22 ± 14.55	875.55 ± 14.68	836.33 ± 15.29		0.062
Model 2	837.45 ± 14.69	873.18 ± 14.44	827.73 ± 15.27		0.086
**RMR per BMI (kcal)**					
Crude	50.65 ± 8.75	51.01 ± 8.54	52.64 ± 9.24	0.275	
Model 1	52.46 ± 1.01	50.15 ± 1.02	50.59 ± 1.06		0.267
Model 2	52.31 ± 1.02	50.74 ± 1.01	50.10 ± 1.06		0.320
**RMR per FFM (kcal)**					
Crude	33.33 ± 4.35	33.99 ± 4.93	34.28 ± 4.10	0.335	
Model 1	34.42 ± 0.54	32.58 ± 0.55	33.55 ± 0.57		0.065
Model 2	33.01 ± 0.54	34.33 ± 0.53	33.18 ± 0.57		0.204

One way ANCOVA.

^*a*^Mean ± SD was presented.

*p for ^b^Mean ± SE was presented and adjusted for energy intake, age, job status, supplement intake, physical activity, BMI.

*P*-value < 0.05 was significant with bold font.

*p ^c^Mean ± SE was presented and adjusted for model 1 + legumes and vegetables.

### Association between the resting metabolic rate and related variables and NOVA score

The relationship between the RMR and NOVA score is shown in Table 4. No significant relationship of RMR variables were observed among NOVA groups in crude models. In model 1, a negative association between the RMR per BMI (β = −0.012, 95% CI: −0.022 to −0.002, *P* = 0.038) with the NOVA score was observed. This negative relationship remained after adjusting for all confounders in model 2. In addition, a negative association was appeared between the NOVA score and RMR (β = −0.15, 95% CI: −0.471 to -0.052, *P* = 0.04), and the RMR per FFM (β = −0.24, 95% CI: −0.006 to -0.000, *P* = 0.04) in model 2.

**TABLE 4:** Association between resting metabolic rate and related variables and UPF intakes.

Variables	β*	95% CI	*P*-value
**RMR (kcal)**			
Crude	0.156	−0.100−0.411	0.231
model 1	−0.184	−0.000 to −0.000	0.054
model 2	−0.159	−0.471 to −0.052	**0.044**
**RMR deviation (kcal)**			
Crude	0.004	−0.009 to 0.017	0.529
Model 1	−0.002	−0.018 to 0.015	0.842
Model 2	−0.006	−0.023 to 0.010	0.454
**RMR per BSA (kcal)**			
Crude	0.036	−0.077−0.149	0.531
Model 1	−0.019	−0.166−0.128	0.799
Model 2	−0.061	−0.208−0.085	0.412
**RMR per BMI (kcal)**			
Crude	0.003	−0.005−0.012	0.462
Model 1	−0.012	−0.022 to −0.002	**0.038**
Model 2	−0.014	−0.025 to −0.006	**0.036**
**RMR per FFM (kcal)**			
Crude	0.003	−0.002−0.007	0.265
Model 1	−0.121	0.000−0.000	0.059
Model 2	−0.241	−0.006 to −0.000	**0.041**

All values were presented as 95% Confidence intervals (95% CI).

*P*-value < 0.05 was significant with bold font. UPF, ultra-processed foods; RMR, resting metabolic rate; BSA, body surface area; BMI, body fat mass; FFM, free fat mass.

β* regression coefficients refer to the UPF tertiles relationship.

Model 1: Adjusted for age, energy intake, BMI, physical activity, job status, and supplement intake.

Model 2: Adjusted for model 1 + legumes and vegetables intake.

### The correlation between inflammatory markers and the resting metabolic rate variables

The correlation between inflammatory markers and the RMR variables is shown in Supplementary Table 2. hs-CRP had a weak positive linear relationship with the RMR (*r* = 0.146, *P* = 0.024) and RMR per FFM (*r* = 0.128, *P* = 0.048), respectively.

### Assessing the mediating role of inflammatory markers between ultra-processed foods intake and resting metabolic rate

The mediatory role of inflammatory markers on the relationship between UPF intakes and RMR is shown in Supplementary Figure 1. The association between the NOVA score and RMR (*P*-value _*primary*_ = 0.04), RMR per BMI (*P*-value _*primary*_ = 0.03), and RMR per FFM (*P*-value _*primary*_ = 0.04) is shown in Table 4. After adjusting for all confounders (Table 5), it was evident that all of the markers may inversely mediate the relationship between the mentioned variables and NOVA scores. Furthermore, by entering hs-CRP (*P*-value _*after*_ = 0.54), IL-1β (*P*-value _*after*_ = 0.42), MCP-1(*P*-value _*after*_ = 0.93), and PAI-1(*P*-value _*after*_ = 0.43) as confounding variables, along with the other confounders in model 2, a potential negative relationship between the NOVA score and RMR per BSA was evident. In addition, hs-CRP (*P* = 0.564) and MCP-1 (*P* = 0.911) also had a possible negative mediatory role in the relationship between NOVA score groups and RMR deviation from normal.

**TABLE 5:** The association of the mediating effect of inflammatory markers on RMR subcategories between UPF tertiles in overweight and obese women.

	NOVA screener			
RMR variables	Inflammatory markers	β*	95% CI	*P*-value
RMR (kcal)	PAI-1	−0.190	−0.643 to 0.264	0.409
	IL-1	−0.363	−1.070 to 0.344	0.305
	hs-CRP	−0.110	−0.433 to 0.212	0.500
	MCP-1	−0.029	−0.369 to 0.311	0.866
RMR deviation (kcal)	PAI-1	−0.011	−0.035 to 0.014	0.384
	IL-1	−0.019	−0.061 to 0.023	0.369
	hs-CRP	−0.005	−0.023 to 0.013	0.564
	MCP-1	−0.001	−0.020 to 0.018	0.911
RMR per BSA (kcal)	PAI-1	−0.088	−0.310 to 0.134	0.435
	IL-1	−0.147	−0.517 to 0.223	0.426
	hs-CRP	−0.049	−0.211 to 0.112	0.547
	MCP-1	−0.006	−0.172 to 0.159	0.939
RMR per BMI (kcal)	PAI-1	−0.006	−0.022 to 0.009	0.434
	IL-1	−0.012	−0.036 to 0.013	0.331
	hs-CRP	−0.004	−0.014 to 0.007	0.516
	MCP-1	−0.001	−0.012 to 0.011	0.910
RMR per FFM (kcal)	PAI-1	−0.004	−0.013 to 0.005	0.399
	IL-1	−0.002	−0.014 to 0.011	0.790
	hs-CRP	−0.001	−0.007 to 0.005	0.719
	MCP-1	−2.326	−0.006 to 0.006	0.994

UPF, ultra-processed foods; RMR, resting metabolic rate; BSA, body surface area; FFM, fat free mass; BMI, body mass index; hs-CRP, high-sensitive C-reactive protein; IL-1, interleukin 1; PAI-1, plasminogen activator inhibitor-1; MCP-1, monocyte chemoattractant protein-1.

Variables were adjusted for age, energy, BMI, physical activity, job status and supplement intake, legumes and vegetables intake.

Logistic regression was used; β* regression coefficients refer to UPF groups.

All values were presented as 95% Confidence intervals (95% CI).

## Discussion

To the author’s knowledge, this is the first study to have investigated the possible mediatory role of inflammatory markers on the relationship between UPF and RMR in obese and overweight women. In this study, we found that with increasing NOVA scores, RMR deviation from the calculated normal RMR was increased, which indicates that with increasing UPF consumption, RMR decreased. In addition, a significant negative association between the RMR, RMR per BMI, and RMR per FFM, with NOVA score was observed after adjusting all confounders. This suggests that all the included markers may inversely mediate the relationship between the mentioned variables and NOVA score. In addition, hs-CRP and MCP-1 also had a possible negative mediatory role in the relationship between NOVA score groups and RMR deviation from normal.

High levels of CRP indicate low-grade inflammation that is, often, associated with several non-communicable diseases, including obesity, cardiovascular disease (CVD), and diabetes (34–37). In a study of 670 adolescent girls in Iran, it was found that there is a positive and significant relationship between adherence to a Western diet and higher serum levels of CRP (38). Western diets contain high amounts of refined grains, snacks, red meats and organ meat, pizza, fruit juices, industrial compote, mayonnaise, sugar, soft drinks, sweets, and desserts. On the other hand, the consumption of fruits and vegetables is inversely correlated with CRP levels, which is comparable to the findings in our study, where fruit and vegetable intake decreased with increasing NOVA scores, and the amount of hs-CRP increased (38). Findings of Lopes et al. suggest that a positive association between excess intake of UPFs and CRP levels in women may be mediated by adipocytes (39). The positive association between the consumption of UPFs and the CRP seen in women may be partly explained by the greater accumulation of body fat in women as BMI is strongly associated with CRP levels in women (40, 41). The consumption of UPFs is known to lead to the overproduction of reactive oxygen species and thereby enhances inflammation by increasing hs-CRP levels and decreasing adiponectin (42–44); indeed, the mechanism that may justify the mediatory role of hs-CRP on the relationship between UPFs and RMR may be related to insulin. In the presence of obesity, CRP levels increase (45, 46), and elevated levels are associated with inflammation and insulin resistance (46). However, insulin sensitivity is inversely related to the RMR (47), which indicates that with consumption of UPFs, hs-CRP levels, inflammation, and insulin resistance increase, which has a decreasing effect on the RMR.

In a group of healthy individuals with normal weight and overweight, the association between RMR and hs-CRP levels, indicative of inflammation, was investigated, and the authors reported that sex significantly affects the age-related changes in body composition, in addition to changes in body composition–REE relationship (48). From a biological perspective, IL-6, which is expressed in adipose tissue, stimulates the production of CRP in the liver and leads to higher levels of circulating hs-CRP. In addition, adipocytes produce and secrete fewer pro-inflammatory and anti-inflammatory cytokines, especially enlarged adipocytes, which may be associated with impaired glucose–insulin homeostasis and impaired energy metabolism (49–52). The products secreted by adipose tissue affect systemic metabolism by inflammatory cytokines, leptin, and PAI-1 (53). In fact, some of the nutritional properties of UPFs, such as high energy density, high glycemic load, and high content of saturated and trans fats, may stimulate inflammation by increasing oxidative stress (39). The results of Yunsheng et al. show that dietary fiber may have protective effects against hs-CRP (54), while in a recent review article (55), King et al. suggested that dietary fiber reduced lipid oxidation, which, in turn, was associated with reduced inflammation. Dietary fiber also helps maintain a healthy gut environment and natural flora, which help prevent inflammation (55); indeed, in our study, with the increasing NOVA score and increasing CRP, the amount of dietary fiber intake decreased.

The study by Bibiloni et al. showed an association between omega-3 and PIA-1 PUFAs in healthy women (56). PUFA intake in the Western diet mainly includes n-6 PUFA (mainly linoleic acid and arachidonic acid), with a ratio of n6:n-3 ranging from 10 to 20:1 (57). Arachidonic acid (AA) is released from the membrane through the lipoxygenase pathway to pro-inflammatory eicosanoids, which are involved in inflammatory activation (58). When n-3 PUFA intake is higher, eicosapentaenoic acid (EPA) and docosahexaenoic acid (DHA) partially replace AA in the cell membrane; thus, fewer biologically active substances are formed, and the balance of n-6 and n-3 eicosanoids changes to compounds with less inflammatory activity (59). Another study, by Miller and colleagues, showed that total fiber intake was inversely related to PAI-1, while insoluble fiber was inversely associated with PAI-1 and MCP-1 in overweight women (60). In our study, with the increasing NOVA score, the intake of linolenic acid and dietary fiber decreased, and the amount of hs-CRP increased, although no significant difference was seen in MCP-1 and PIA-1. The mechanism of the mediatory role of MCP-1, like hs-CRP, may also be related to insulin secretion and resistance. MCP-1 secretion is stimulated by tumor necrosis factor α (TNFα), IL-6, and IL-1β, which are secreted from adipose tissue (61, 62). Elevated MCP-1 levels are associated with insulin resistance and type 2 diabetes (T2DM) (63, 64), and as noted earlier, insulin resistance is inversely related to RMR (47). However, MCP-1 and hs-CRP had mediating effects on the association between RMR and UPF, which could be due to differences in the rate of inflammation and fiber intake (total, soluble, and insoluble), as compared with previous studies.

In a randomized controlled trial performed by Hall et al., it was reported that energy intake was higher during adherence to a UPF diet (16). Participants consumed more carbohydrates and fats and gained weight and body fat. The authors also showed that eating UPFs reduced the secretion of the hunger hormone ghrelin and also increased the level of the satiety hormone PYY (YY peptide) (11). Thus, UPFs may more efficiently regulate and stimulate biological mechanisms of hunger and satiety control than processed foods. The authors also asserted that with the unprocessed diet, a decrease in the inflammatory biomarker of hs-CRP was observed and that inflammation may be associated with satiety signals in animal studies (65).

Evidence is emerging regarding the mechanisms that strengthen the link between UPFs and adverse health outcomes. The mechanisms proposed are as follows: poor nutritional profile, UPFs are rich in sodium, added sugars, trans fats, and replace unprocessed foods in the diet (66–70), decrease in intestinal and brain satiety signal due to changes in physical properties caused by food processing and higher glycemic load (71–74), carcinogens formed during high-temperature cooking (acrylamide) (75, 76), and inflammatory responses associated with cellular nutrients and industrial food additives, increased intestinal permeability, and dysbiosis of the intestinal microflora (65, 77, 78).

Carbohydrate metabolism is more energy-intensive than fat metabolism, while protein metabolism requires the most energy (79–81). Processed foods have lower nutrient densities (less content and variety of nutrients per calorie) than whole foods, where extra simple carbohydrates (82–84) and less dietary fiber that make them chemically and structurally simple and digestible (82, 83, 85). All these reduce the volume of meals and reduce satiety, which consequently lead to increased daily caloric intake (31–39), which is associated with obesity and systemic inflammation (86–88).

The strengths of this study are the use of an FFQ questionnaire, which has been specifically validated in the Iranian population. Nevertheless, residual confounding related to recall bias must be acknowledged. Examining this association in one gender and only in Tehran represent limitations of this study because these results cannot be extrapolated to the entire Iranian population and all individuals. The cross-sectional design of this study precludes causal inferences being made. In addition, using BIA may overestimate lean body mass.

## Conclusion

Ultra-processed foods may be related with changes in the production and secretion of cytokines and inflammatory factors and, ultimately, cause inflammation and reduced RMRs. A negative association between the RMR, RMR per BMI, and RMR per FFM with the NOVA score was observed in the present study. UPF intake is likely related with the RMR, RMR per BMI, and RMR per FFM, mediated by the production of hs-CRP, PAI-1, MCP-1, and IL-1β. hs-CRP and MCP-1 levels also had a possible negative mediatory role in the relationship between NOVA score groups and RMR deviation from normal. Given the novel evidence provided, further work, in the form of interventional studies, is needed in this area.

## Data availability statement

The original contributions presented in this study are included in the article/Supplementary material, further inquiries can be directed to the corresponding author.

## Ethics statement

The studies involving human participants were reviewed and approved by the Tehran University of Medical Sciences and Health Services. The patients/participants provided their written informed consent to participate in this study.

## Author contributions

NB and FS designed the project. FS collected the samples and analyzed the data. NB and SN wrote the manuscript. FS, CC, and NB reviewed and edited the manuscript. KM conducted the research and had primary responsibility for the final content. All authors read and approved the final manuscript.

## Supplementary material

Supplementary Figure 1 The mediatory role of inflammatory markers on the relationship between UPF intakes and RMR.

## Abbreviations

RMR: resting metabolic rate
LED: low energy density
HED: high energy density
UPFs: ultra-processed foods
PA: physical activity
WC: waist circumference
WHR: waist-to-hip ratio
BMI: body mass index
PAI-1: plasminogen activator-1
IL-6: interleukin-6
PUFA: polyunsaturated fatty acids
MCP-1: monocyte chemoattractant protein
hs-CRP: high-sensitivity C-reactive protein
HDL-C: high-density lipoprotein cholesterol
FBS: fasting blood sugar
LDL-C: low-density lipoprotein cholesterol
TC: total cholesterol
TG: triglyceride
IC: indirect calorimetry
BSA: body surface area
FFM: free fat mass
FFQ: food frequency questionnaire
IPAQ: the International Physical Activity Questionnaire
METs: metabolic equivalents
ANOVA: one-way analysis of variance
ANCOVA: analysis of covariance.
