# Supplementary material for: Is there any putative mediatory role of inflammatory markers on the association between ultra-processed foods and resting metabolic rate?
Source: Front Nutr. 2022 Oct 13;9:932225. doi: 10.3389/fnut.2022.932225 (PMC9606709; doi:10.3389/fnut.2022.932225)
Supplement: Supplementary Figure 1 — The mediatory role of inflammatory markers on the relationship between UPF intakes and RMR. [file Presentation_1.PPTX]

## Slide 1
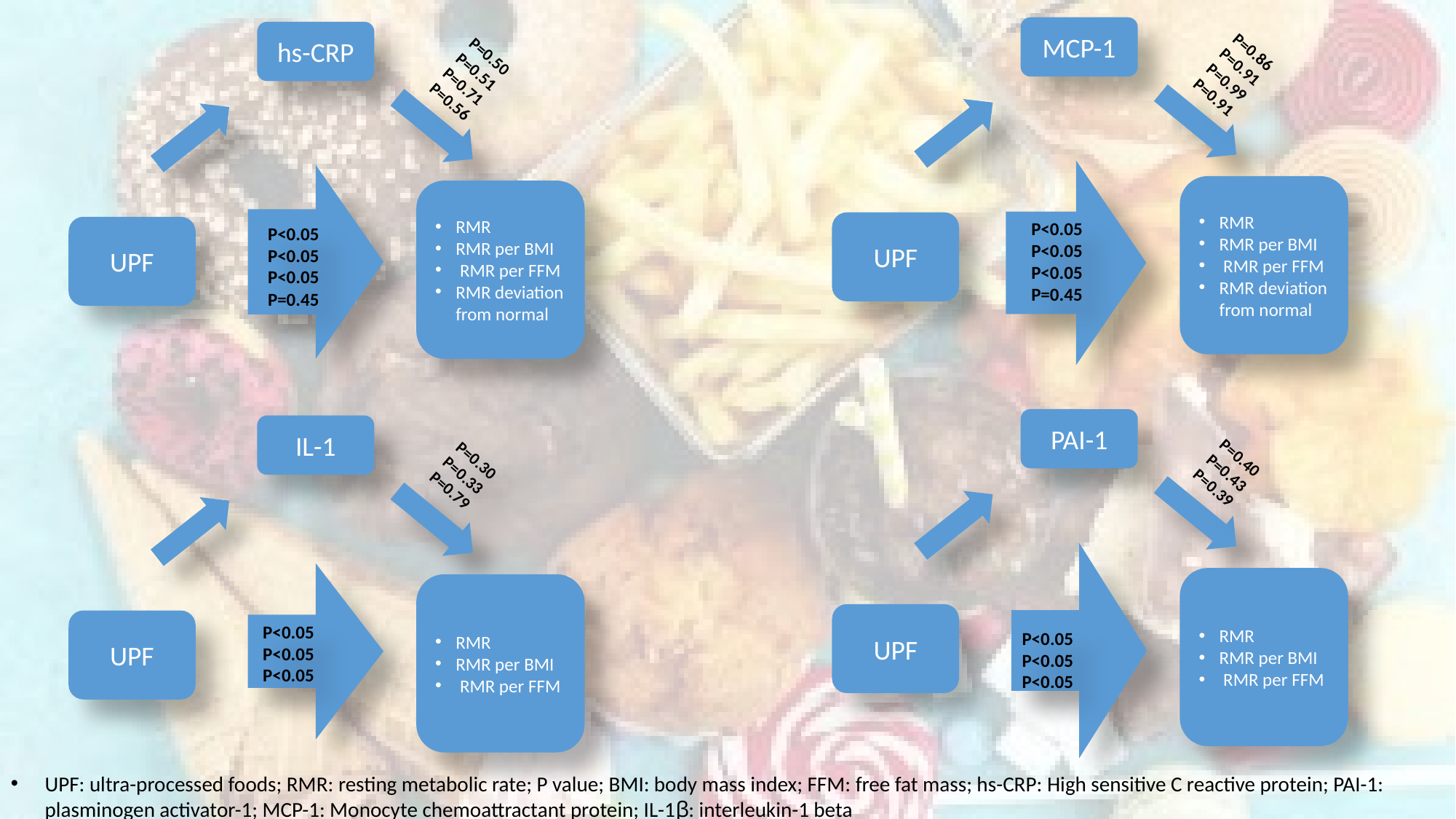

MCP-1
hs-CRP
P=0.86
P=0.91
P=0.99
P=0.91
P=0.50
P=0.51
P=0.71
P=0.56
RMR
RMR per BMI
 RMR per FFM
RMR deviation from normal
RMR
RMR per BMI
 RMR per FFM
RMR deviation from normal
P<0.05
P<0.05
P<0.05
P=0.45
UPF
P<0.05
P<0.05
P<0.05
P=0.45
UPF
PAI-1
IL-1
P=0.40
P=0.43
P=0.39
P=0.30
P=0.33
P=0.79
RMR
RMR per BMI
 RMR per FFM
RMR
RMR per BMI
 RMR per FFM
UPF
UPF
P<0.05
P<0.05
P<0.05
P<0.05
P<0.05
P<0.05
UPF: ultra-processed foods; RMR: resting metabolic rate; P value; BMI: body mass index; FFM: free fat mass; hs-CRP: High sensitive C reactive protein; PAI-1: plasminogen activator-1; MCP-1: Monocyte chemoattractant protein; IL-1ꞵ: interleukin-1 beta
