# Supplementary material for: Is there any putative mediatory role of inflammatory markers on the association between ultra-processed foods and resting metabolic rate?
Source: Front Nutr. 2022 Oct 13;9:932225. doi: 10.3389/fnut.2022.932225 (PMC9606709; doi:10.3389/fnut.2022.932225)
Supplement: Supplementary file 2 [file Data_Sheet_1.docx]

| **SUPPLEMENTARY Table 1 Dietary share (%) of each UPF (% of total UPF) included in the Nova screener for the consumption of ultra-processed foods** | |
| --- | --- |
| **UPF** | **%** |
| **Nondairy beverages** | 39.12 |
| **Cookies-cakes** | 22.63 |
| **Dairy beverages** | 11.00 |
| **Potato chips-salty snacks** | 5.09 |
| **Processed meat-fast foods** | 9.20 |
| **Oil-sauces** | 4.33 |
| **Sweets** | 8.61 |
| **Abbreviation: UPF: Ultra-processed foods** | |

| **SUPPLEMENTARY Table 2 the correlation between inflammatory markers and the RMR variables** | | | | | | | | |
| --- | --- | --- | --- | --- | --- | --- | --- | --- |
| **Variables ^a^** | **PAI-1** | | **IL-1** | | **hs-CRP** | | **MCP-1** | |
|  | **R** | **P** | **R** | **P** | **R** | **P** | **R** | **P** |
| **RMR(kcal)** | 0.008 | 0.913 | 0.106 | 0.360 | 0.146* | **0.024** | -0.120 | 0.067 |
| **RMR deviation (kcal)** | -0.036 | 0.644 | 0.064 | 0.586 | 0.004 | 0.950 | -0.097 | 0.143 |
| **RMR per BSA (kcal)** | -0.029 | 0.705 | 0.074 | 0.523 | 0.053 | 0.418 | -0.075 | 0.256 |
| **RMR per BMI (kcal)** | -0.067 | 0.386 | 0.080 | 0.490 | -0.101 | 0.120 | -0.079 | 0.231 |
| **RMR per FFM (kcal)** | -0.077 | 0.319 | 0.071 | 0.543 | 0.128^*^ | **0.048** | -0.092 | 0.164 |
| ***Abbreviations: SAAs: sulfur amino acids, WHtR: waist to height, BMI: body mass index, WC: waist circumference***  **** Significant relationship less than 0.05***  ***** Significant relationship less than 0.01***  ***^a^ variables were adjusted for energy intake.***  ***Analyzes were performed based on Spearman correlation test.*** | | | | | | | | |
